# Updates to HCOP: the HGNC comparison of orthology predictions tool

**DOI:** 10.1093/bib/bbab155

**Published:** 2021-05-06

**Authors:** Bethan Yates, Kristian A Gray, Tamsin E M Jones, Elspeth A Bruford

**Affiliations:** HUGO Gene Nomenclature Committee (HGNC), European Molecular Biology Laboratory, European Bioinformatics Institute, Wellcome Genome Campus, Hinxton, Cambridge CB10 1SD, UK; HUGO Gene Nomenclature Committee (HGNC), European Molecular Biology Laboratory, European Bioinformatics Institute, Wellcome Genome Campus, Hinxton, Cambridge CB10 1SD, UK; HUGO Gene Nomenclature Committee (HGNC), European Molecular Biology Laboratory, European Bioinformatics Institute, Wellcome Genome Campus, Hinxton, Cambridge CB10 1SD, UK; HUGO Gene Nomenclature Committee (HGNC), European Molecular Biology Laboratory, European Bioinformatics Institute, Wellcome Genome Campus, Hinxton, Cambridge CB10 1SD, UK; Department of Haematology, University of Cambridge School of Clinical Medicine, Cambridge Biomedical Campus, Cambridge CB2 0AW, UK

**Keywords:** nomenclature, orthologs, vertebrates, aggregation, meta-database, website

## Abstract

Multiple resources currently exist that predict orthologous relationships between genes. These resources differ both in the methodologies used and in the species they make predictions for. The HGNC Comparison of Orthology Predictions (HCOP) search tool integrates and displays data from multiple ortholog prediction resources for a specified human gene or set of genes. An indication of the reliability of a prediction is provided by the number of resources that support it. HCOP was originally designed to show orthology predictions between human and mouse but has been expanded to include data from a current total of 20 selected vertebrate and model organism species. The HCOP pipeline used to fetch and integrate the information from the disparate ortholog and nomenclature data resources has recently been rewritten, both to enable the inclusion of new data and to take advantage of modern web technologies. Data from HCOP are used extensively in our work naming genes as the Vertebrate Gene Nomenclature Committee (https://vertebrate.genenames.org).

## Background

Orthologs are homologous genes in different species that have originated through speciation from a single gene in a common ancestor. The rapid expansion in the number of genomes available over recent years means identifying orthologous genes between species is increasingly important for genome annotation, as it enables the inference of the possible function of a gene based on the previously reported function of its orthologs.

Multiple specialized tools, each utilizing different methodology and covering a different taxonomic range of species, have been published that predict orthology assertions. As a general rule, these resources input coding sequence data from multiple species and produce orthology assertions as output. Since each resource was created with different evolutionary questions and goals in mind, these can differ significantly in methodology, input data and output formats. Existing resources employ a variety of computational methods including (but not limited to) BLAST [1] alignments, Hidden Markov Models [2] and phylogenetic analyses in order to make orthology assertions. Many resources use a combination of different methods to produce their orthology inferences. In addition to differences in methodology, there is also considerable variety in the input data for each resource, such as the number and evolutionary diversity of represented species, or the way that coding sequences are predicted for a given species’ genome. Most resources get their source data from either Ensembl [3] or NCBI [4]; many also use species-specific data from model organism databases (MODs), which may include manual curation in addition to the Ensembl/NCBI gene models. Other orthology resources use UniProt [5] proteomes as their initial data source. For source data that have periodic releases such as Ensembl, there are also considerable differences in the release versions of the data used, both between resources and for different species included in a single resource. Finally, different resources output and disseminate their data in a wide range of formats and venues. Some resources are integrated into larger biological databases, while others exist as standalone tools, and the orthology data may be made available in varying file formats. This underlying diversity in method and in output both inevitably lead to differences in each resource’s results and also make it time consuming and potentially complicated for a user to compare and contrast the orthology assertions from different resources.

The HGNC Comparison of Orthology Predictions search tool [6] (HCOP, https://www.genenames.org/tools/hcop) was created in the early 2000s to aggregate, display and simplify searching of orthology assertions from a variety of orthology prediction tools, for a specified human gene or set of genes. Since its original inception, HCOP has undergone a series of changes as new orthology resources were published and computer technologies changed, culminating in a complete reimplementation of the HCOP pipeline to better utilize the available data and computational methods. The changes made to HCOP include the addition of several new species, increasing from just 4 species in 2006 to a total of 20 in 2021. If a species has its own nomenclature committee or MOD, the gene nomenclature data shown in HCOP for that species are now imported directly from them. The number of orthology sources aggregated by HCOP has increased to 14 from an initial 6, with some of the original orthology sources being removed due to the resource no longer being maintained or updated. The decision to add a new orthology source involves several factors, including the availability of the orthology data, update frequency, species coverage and methods used to create the orthology assertions.

Originally HCOP combined assertions by mapping the orthology source data, which often report orthologies using a protein identifier, to an NCBI Gene ID [7] and creating pairs of NCBI Gene IDs. This approach was problematic as it would miss some orthology assertions, for example where it was not possible to link the source data to an NCBI Gene ID, either because NCBI Gene had not annotated a corresponding gene model or because the correct ID was difficult to map computationally. HCOP has since been modified to allow for the mapping of the orthology source data to either a NCBI Gene ID or an Ensembl gene ID and additionally generates an equivalency between NCBI Gene and Ensembl gene IDs where these both exist allowing HCOP orthology assertions to be represented as pairs of NCBI Gene–Ensembl Gene ID combinations; for example, HCOP represents the mouse ortholog assertion to human *BRCA1* gene as being ‘12189-ENSMUSG00000017146’ to ‘672-ENSG00000012048’. This change results in fewer lost assertions as only one of Ensembl or NCBI needs to have annotated a corresponding gene model.

The large increase in the amount of data used to build HCOP, combined with the availability of modern computational methods, made rewriting the data production pipeline necessary. The new pipeline has been developed in a modular manner and can be run on a high-performance computing cluster allowing for precise control and parallelization of the individual processes. Pipeline runtime is much reduced allowing for daily rather than weekly update of the HCOP data.

As part of the new implementation of HCOP, the database used to store the data was changed from PostgreSQL to MySQL, to support easier inclusion of the HCOP data within the genenames.org website, which is backed by its own MySQL database. The interface for the HCOP web tool has also been modernized as part of a larger redesign of the genenames.org website. These changes are reported in more detail below.

HCOP currently aggregates information from eggNOG [8], Ensembl Compara [9], HGNC [10], HomoloGene [11], Inparanoid [12], OMA [13], OrthoDB [14], OrthoMCL [15], NCBI Orthologs [11], Panther [16], PomBase [17], PhylomeDB [18], TreeFam [19] and The Zebrafish Information Network (ZFIN) [20] into a single tool, allowing easy comparison of these disparate data to identify a consensus orthology prediction. HCOP has been a member of the Quest for Orthologs Consortium [21] since 2009. While HCOP was originally developed to compare predicted orthologs between human (*Homo sapiens*) and mouse (*Mus musculus*), it has since been expanded to predict orthologs between human and chimp (*Pan troglodytes*), macaque (*Macaca mulatta*), rat (*Rattus norvegicus*), dog (*Canis lupus familiaris*), cat (*Felis catus*), horse (*Equus caballus*), cow (*Bos taurus*), pig (*Sus scrofa*), opossum (*Monodelphis domestica*), platypus (*Ornithorhynchus anatinus*), chicken (*Gallus gallus*), anole lizard (*Anolis carolinensis*), xenopus (*Xenopus tropicalis*), zebrafish (*Danio rerio*), *Caenorhabditis elegans*, fruit fly (*Drosophila melanogaster*), *Saccharomyces cerevisiae* and *Schizosaccharomyces pombe.* As each individual orthology resource selects its own set of taxa to predict ortholog assertions for, not every resource will have data for each of the species in HCOP. Table 1 lists the orthology resources, the species the resource covers and the current version of the data in HCOP.

**Table 1:** Orthology sources in HCOP

Orthology source	Version	Species data applies to
eggNOG	Version 5.0	All species except horse
Ensembl	Release 102	All species
HGNC	N/A	Human and mouse
HomoloGene	Release 68	Human, chimp, macaque, mouse, rat, dog, cow, chicken, xenopus, zebrafish, *C. elegans,* fruit fly, *S. cerevisiae* and *S. pombe*
InParanoid	Version 8.0	All species
OMA	Release January 2020	All species
OrthoDB	Version 10.1	Human, chimp, macaque, mouse, rat, dog, cat, horse, cow, pig, opossum, platypus, chicken, anole lizard, xenopus, zebrafish, *C. elegans* and fruit fly
OrthoMCL	Version 5	Human, mouse, dog, chicken, zebrafish*, C. elegans*, fruit fly, *S. cerevisiae* and *S. pombe*
NCBI Gene orthologs	N/A	All species except *C. elegans*, fruit fly, *S. cerevisiae* and *S. pombe*
Panther	Version 15	All species
PhylomeDB	Version 4, data are taken from phylome 514	Human, chimp, macaque, mouse, rat, dog, cow, opossum, platypus, chicken, xenopus, zebrafish, *C. elegans*, fruit fly, *S. cerevisiae* and *S. pombe*
PomBase	N/A	Human and *S. pombe*
TreeFam	Release 9.0	All species except cat, *S. cerevisiae* and *S. pombe*
ZFIN	N/A	Human and zebrafish

In addition to orthology data, HCOP also imports further data from HGNC, VGNC [10], Mouse Genome Informatics (MGI) [22], Rat Genome Database (RGD) [23], Chicken Gene Nomenclature Consortium (CGNC) [24], Xenbase [25], ZFIN [20], WormBase [26], Saccharomyces Genome Database (SGD) [27] and PomBase [17] to ensure that the current approved gene symbols, names, locus types and location information from the appropriate nomenclature or MOD are displayed. For those species without an official nomenclature committee, or where a nomenclature committee exists but gene data are not available programmatically, this information is taken from the NCBI Gene database or from Ensembl if the gene in question cannot be mapped to an NCBI Gene identifier.

## The HCOP pipeline

The HCOP pipeline code that combines the data from each of the orthology and nomenclature sources was recently completely rewritten to utilize the eHive production system [28] developed by the Ensembl project. Using eHive allowed for the modularization of the code, giving precise control over how and when each data source gets updated, as well as enabling parallelization of the individual update processes. All source data used to build HCOP are stored in a MySQL database, along with metadata generated each time the pipeline runs that records the version of the data and when it was last updated for each of the species it relates to. Data can be selectively updated, both in terms of the resources and the species or set of species.

**Figure 1:**
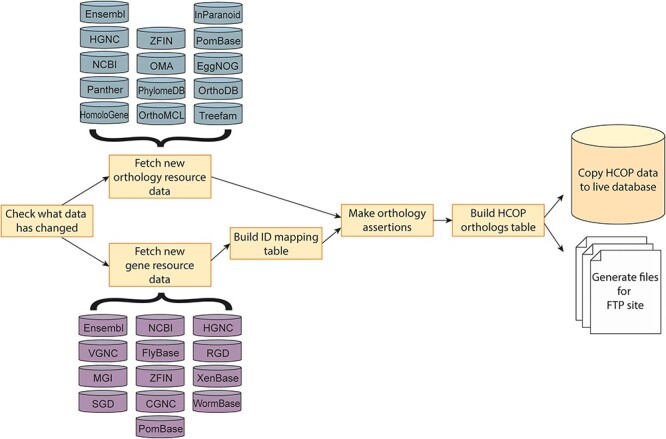
The HCOP data production pipeline. The pipeline is broken up into several stages: nomenclature/gene resource data update, orthology data update, generation of an ID mapping table which links together the MOD ID, NCBI Gene ID, Ensembl stable gene ID and UniProt identifiers for a specific gene, conversion of the raw orthology data to HCOP orthology assertions which include the appropriate gene ID information from the ID mapping table and finally combination of assertions that share the same NCBI Gene and Ensembl gene IDs to produce a single combined ortholog for each ortholog pair. Each combined ortholog is added to the HCOP orthologs table in the HCOP MySQL database, which is then used to update the public database and FTP site files.

Figure 1 shows how the pipeline is broken up into the following stages: nomenclature data update, orthology data update, generation of an ID equivalency table to determine which MOD ID, NCBI Gene ID, Ensembl stable gene ID and UniProt identifiers relate to a specific gene, conversion of the raw orthology data to HCOP orthology assertions that include the corresponding NCBI Gene and Ensembl gene IDs as specified in our equivalency table and finally combination of assertions that share the same NCBI Gene and Ensembl gene IDs. It is these combined ortholog predictions, essentially a pair of genes with a list of associated databases that support that orthology assertion, that are displayed as HCOP results.

Calculating ID equivalency is a multi-step process run using SQL queries and differs between species depending on whether or not there is a MOD for that particular species. Each gene is added to an ID mapping table, which contains the taxon ID, Ensembl ID, NCBI Gene ID, MOD ID, UniProt ID and gene symbol. MySQL coalesce queries prioritize ID mapping information provided from the MOD (where applicable), then Ensembl and finally NCBI Gene. Once this process has been completed for all genes in the species, any duplicate rows in the table are removed.

Prior to running each nomenclature and orthology update, the pipeline first completes checks to work out if the relevant data have altered since it was last imported, and only runs the update step in the event of a new data release or version. If any of the nomenclature data processes have been run, the pipeline determines which species have updated nomenclature data and runs the step to regenerate the ID equivalency data for those particular species. In the event of orthology data being updated, the steps to produce equivalent HCOP orthology assertions for that source will also need to be run, along with the steps that combine the orthology assertions for any affected species. In the event of the ID equivalency data being updated for a species, all of its corresponding orthology assertions from each ortholog source will be regenerated—this ensures that HCOP always displays the latest nomenclature data for a given gene in its results.

## Querying HCOP

In late 2018, a new version of the https://www.genenames.org website that was built using Angular.js and the Bootstrap CSS framework was released. This new website incorporated changes to HCOP both in terms of the interface used for query submission and in the way a query submission is processed. The HCOP interface is split into three components: the search form, bulk downloads and results. If a query has not yet been submitted, the users will see only the first two of these components.

The species of the query gene can be selected using the dropdown menu in the ‘Search for ortholog(s) between’ section of the form, and one or more species to query for potential orthologs are selected using the target species checkboxes just below this. By default, the interface selects human as the query species and all species within HCOP are queried. If the query input gene is not from human, HCOP will only identify human orthologs of that gene. It is possible to specify the orthology sources represented in the results by selectively checking the checkboxes in the ‘include orthologs from’ section of the form. If a certain source is excluded from a query, any ortholog assignments based solely on that source will not appear in the result set. If, however, the orthology assignment is supported by one or more of the other ortholog sources selected for inclusion in the search, the excluded source will still be listed as one of the resources supporting that particular assignment. Orthology assertions can be obtained for a given gene by searching with either its Ensembl gene identifier or its NCBI Gene identifier. For species with either a model organism or nomenclature database, it is also possible to search with the approved gene symbol, the approved gene name or the gene identifier from that database. The query type (approved symbol, name or specific database ID) should be selected from the ‘where’ dropdown menu, and the available choices dynamically change depending on the query species. Database identifier prefixes like ‘HGNC:’, ‘VGNC:’, ‘MGI:’, ‘RGD:’, ‘ZFIN’, ‘SGD:’, ‘XENBASE’, ‘BGD:’ or ‘CGNC:’ are not required and a search will work whether they are included or not.

HCOP results for multiple genes can be viewed simultaneously by searching with a list of query terms, separated by commas, newlines or spaces. This list may either be pasted into the ‘enter identifier(s)’ box or uploaded as a file using the ‘upload file’ option on the form. If provided with a list of terms (either comma, space or newline delimited), HCOP searches with each term in turn, e.g. *ABCA1 ABCA2 ABCA3*. Wild cards are supported when the query term is an approved symbol: ‘_’ substitutes for a single character and ‘*’ or ‘%’ substitutes for one or more characters. For example, ABCA* fetches all genes beginning *ABCA*, while *ABCA_* fetches only *ABCA1* to *ABCA9*. It is not advisable to start a query with a wildcard as the search will not be able to utilize any indexed fields in the HCOP database and the search will be slow. Searches are case insensitive so searching *ABCA1* will give the same result as searching *abca1*.

When an HCOP query is submitted, it triggers a JavaScript process, which first determines the type of query being run. If the query term contains a wildcard or if the query type is ‘Approved name contains’, the first step in generating the results is to fetch all the possible query terms represented by that query. A separate request for HCOP results is then made for each distinct query term. The service uses ngQueue, an AngularJS module, which manages the requests being sent and controls the number of concurrent processes being run to prevent the server from being overloaded. Each request for HCOP results is made via a RESTful endpoint, which returns data in JSON format for web display.

## HCOP Results

**Figure 2:**
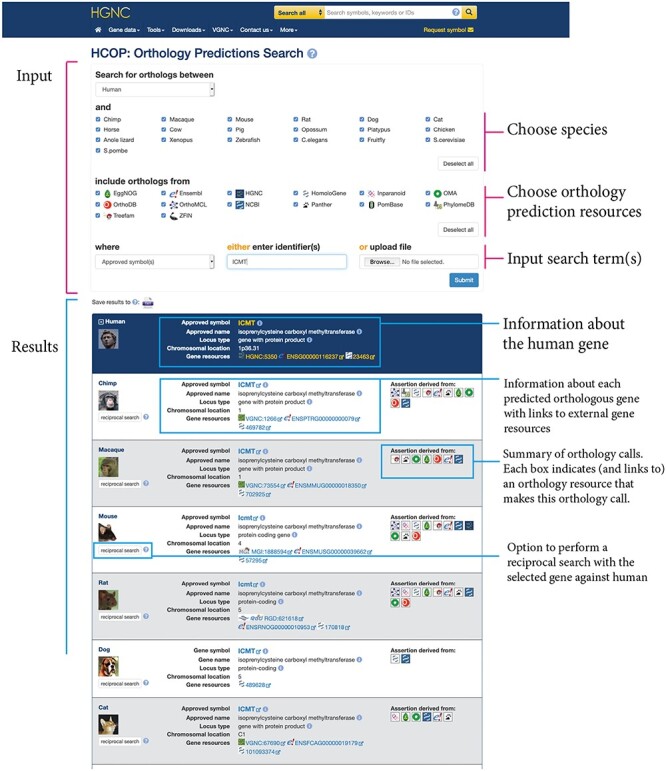
The HCOP web tool interface. The ‘Input’ section shows the updated HCOP search form. The users select a primary species and one or more species that they wish to identify orthologs in. They then select the ortholog resources they wish to include in the search, and the type of search term, e.g. approved symbol, or database identifier, that they are providing. A single search term or list of search terms may be pasted into a text box or uploaded as a file to be used to run the search. The ‘Results’ section shows an example result panel. Information about the query gene appears in the dark blue section at the top of the panel, with each ortholog identified having its own section below this. Basic information about both the query and ortholog genes, as well as links to these genes in other resources, is displayed. Each ortholog section has an additional column labeled ‘Assertion derived from’ that contains a set of icons that represent the orthology sources that support this assignment.

An HCOP result is a mapping between a query gene and its ortholog(s) in the target species. If more than one query term was provided, there will be a separate results panel for each query term that has a matching ortholog assignment in HCOP, and the results sections will be scrollable. Figure 2 shows an example results panel. At the top of the results panel in the dark blue section is data relating to the query term supplied, below this are alternating white and light blue sections, each of which represents an orthology assertion for the query gene. Basic summary information is provided for both the query and its orthologs, including the gene symbol and name, the locus type, chromosomal location and links to other gene resources such as NCBI Gene and Ensembl. The gene symbol and gene name will be prefixed either with ‘Approved’ to indicate that the source of the data is a nomenclature committee or with ‘Gene’ to indicate that the nomenclature data being displayed have been imported from either NCBI Gene or Ensembl. Clicking on the small information icons next to either the symbol or the name displays the origin of the data.

Each orthologous gene also has a section labeled ‘Assertion derived from’ that contains a series of icons. These icons represent the ortholog resources that support the particular ortholog assignment. The higher the number of resources agreeing on a particular assertion the more confidence we can have that this assertion reflects a genuine ortholog. Each icon is a link to the entry for that gene in its corresponding orthology database, and hovering over the icon will display the full name of the ortholog data source. If there are multiple assertions returned for any species, the assertion supported by the most resources is shown first.

As HCOP makes a separate RESTful HTTP request for each query term submitted, each individual result will be displayed as soon as the request returns data to the web server. When all requests have been completed, a link to download the complete result set as a tab-separated text file is displayed above the first result panel.

In addition to being able to download results from a specific query, precomputed files of HCOP data can be downloaded from our FTP site or via the ‘Bulk Downloads’ section of the HCOP tool. The users have the option of retrieving a file containing ortholog data from human and another single species or ortholog data from all HCOP species in a single file. For the human and single ortholog species files, the ‘6 Column’ output returns the raw assertions, Ensembl gene IDs and NCBI Gene IDs for human and one other species, while the ‘15 Column’ output includes additional information such as the chromosomal location, accession numbers and, where possible, references the approved gene nomenclature. The files containing all species ortholog data have an additional column at the start giving the taxon id for each ortholog species.

HCOP results for any named human protein-coding gene are also readily accessible from the gene’s symbol report page on the HGNC website (http://www.genenames.org). These results are displayed in their own tab, labeled ‘HCOP homology predictions’, which provides a quick way for users to see HCOP data without them having to leave the gene symbol report in order to submit a query using the HCOP tool.

Direct comparison of the orthology predictions made by the individual orthology sources included in HCOP is not straightforward due to a large disparity in species coverage, methodologies used and data returned by each of the resources. Several resources (HGNC, PomBase and ZFIN) only report on the relationship between human genes and one other species but include manually curated data and as such represent high confidence predictions. Others such as Ensembl Compara and OMA report orthology as pairwise relationships and provide information as to what type of orthology each pairwise call represents, for example if it is a one-to-one or a one-to-many relationship. Resources such as EggNOG and OrthoMCL aim to identify groups or clusters of orthologous protein sequences and often report multiple potential orthologs for a given gene. Each approach has its benefits, with pairwise results making identification of one-to-one orthologs straightforward and orthologous clusters being useful for exploring gene duplication events and gene family expansions. Many genes where a single human ortholog cannot be identified belong to large and complex gene families, such as olfactory receptors, cytochrome P450s, histones and zinc fingers, where orthology relationships are often harder to determine via automated methods.

A major advantage of HCOP is that it brings all this information together into a single interface, enabling user interpretation of the data. If a human query gene returns more than one potential ortholog, a measure of reliability can be provided by looking at the number of resources that support each prediction. For example if two results were returned, the first of which is only supported by one or even two resources, while the other result is supported by four or more, we would have greater confidence in the second result having a closer relationship to the query gene. Often when an orthology relationship is only supported by a single resource, this will be because the resource focuses on identifying clusters of orthologous protein sequences rather than pairwise assignments. Alternatively, the resource may be using an outdated version of the source data compared to the other resources due to differing update cycles.

Twelve of the orthology resources in HCOP contain human to mouse orthology data; these resources unanimously identify a mouse ortholog for 38% of human protein coding genes, with 5% of these cases identifying more than one putative mouse ortholog. Rat orthology data are present in 10 orthology sources, all of which predict the same ortholog(s) for 21% of human protein coding genes. However, for chicken, which has data from 11 of the orthology sources, only 3% of human genes have an ortholog(s) that is confirmed by all 11 resources. This low concordance is explained by data based on legacy genome assemblies in several resources: it is often not possible to map the protein identifiers used by these resources to the correct gene model in the current chicken assembly. Limiting the chicken orthology data to require 8 of the 11 resources to confirm an orthology relationship identifies an ortholog(s) for 29% of human genes. These figures highlight the diversity in results from each resource and demonstrate that the user must exercise their own judgment when comparing the results from each resource.

## Applications of HCOP data

The Vertebrate Gene Nomenclature Committee (VGNC) was founded in 2016 as an extension of the established HGNC project. VGNC is responsible for assigning standardized names to genes in selected vertebrate species that currently lack a nomenclature committee. The VGNC also coordinates with the five existing vertebrate nomenclature committees, MGNC (mouse), RGNC (rat), CGNC (chicken), XNC (Xenopus frog) and ZNC (zebrafish), to ensure that genes are named in line with their human homologs.

The VGNC project initially began with naming genes in chimp, expanded to include horse, cow and dog, and then added cat, macaque and most recently pig. The VGNC uses a software pipeline that searches the HCOP data to identify a high confidence set of ortholog predictions between human and each of the VGNC species. In order to be considered high confidence, the ortholog assertion must represent a one-to-one orthology relationship between protein coding genes and must be predicted by at least three out of the following four resources: Ensembl, NCBI Gene, OMA and PANTHER. When all four resources confirm an orthology assertion, the equivalent VGNC species gene is automatically assigned the same nomenclature as the human gene, so long as it also passes a series of other rules set by HGNC curators, which have been devised to ensure that the nomenclature is suitable for transfer across vertebrates. Where less than four of the orthology resources confirm an assignment, or if one of the additional curation checks fails, the orthology assignment is flagged for manual curation and will be reviewed by an expert curator prior to its potential inclusion in the VGNC gene set.

All VGNC data are available via a dedicated website https://vertebrate.genenames.org, and the NCBI Gene, Ensembl and UniProt databases now display approved VGNC nomenclature for all relevant entries, along with reciprocal links to the symbol reports on the VGNC site. VGNC is also included in the registry of resources at identifiers.org.

## Comparison with existing orthology aggregator tools

Several other online tools have been developed that also aim to aggregate orthology information. Each of these tools differs in terms of the orthology sources utilized, species coverage and update frequency. Possibly the closest in scope to HCOP is the DIOPT tool [29], but ORCAN [30] and OrthoList 2 [31] are other tools that share some overlapping functionality with HCOP.

The orthology prediction comparison tool DIOPT was first published in 2011. This tool has a similar goal to HCOP in that it provides a way for users to compare a variety of orthology prediction resources and identify consensus orthology calls. Although both HCOP and DIOPT have significant overlap in the orthology prediction resources that are represented, there are some differences between the scope and functionality of these tools that users should be aware of, so that they can select the appropriate tool for their needs. DIOPT contains data for the major laboratory model organisms, mouse (*M. musculus*), rat (*R. norvegicus*), xenopus (*X. tropicalis*), zebrafish (*D. rerio*), *C. elegans*, fruit fly (*D. melanogaster*), *S. cerevisiae*, *S. pombe* and Thale cress (*Arabidopsis thaliana*), as well as human, and can be used to search for orthology assertions between any of those species. Thus, it is most relevant to researchers working on those major model organisms in their own research. In contrast, HCOP was initially designed to be human centric and currently searches for orthologs between human and other species, or vice versa. The species included in HCOP encompass a number of key vertebrate species in addition to major model organisms. HCOP is updated whenever the underlying data are updated, for example when one of the orthology resources creates a new release, whereas DIOPT updates data sources more periodically, historically about every 18–24 months. One of DIOPTs features is providing a score for each putative ortholog found, which reflects the number of resources that retrieve the same orthology assertion. The score contribution from each resource is weighted by factors such as whether the assertion arises from manual curation. One danger with presenting a score is that it can give the user a potentially false sense of confidence if the score is high, which could lead the user to assume that the highest scored assertion is the correct ortholog. Sometimes this will indeed be the case, but it is also possible that a high scoring assertion is based on underlying biases in the data, for example if a gene has been duplicated in one species but only one copy of the duplication has been annotated in that species’ genome, all orthology resources using that annotation set will predict a single ortholog for that species and thus a high score could be generated for that copy and cause a user to believe that this is a one-to-one ortholog. For this reason, HCOP does not assign any type of score to orthology predictions; instead, it presents all orthology assertions for a given query and links out to each orthology prediction resource. This encourages the user to examine each assertion and judge which ortholog prediction(s) they are most confident in, given the underlying data.

ORCAN is an online tool that aims to provide evolutionary and functional annotation of protein sequences. As part of the evolutionary analysis step, it directly queries five orthology databases, as well as running four orthology prediction tools. ORCAN differs significantly from HCOP: rather than utilizing precalculated orthology data, all analysis steps are performed on the fly with the tools that run directly using the most recent UniProt complete proteome set. This enables the use of the latest available data but the trade-off to this is it can take several minutes to complete all of the steps required, with the results of individual processes appearing only when they are complete. In addition to identifying potential orthologs, other results returned include information on protein domain organization, sequence modification, Gene Ontology (GO) terms [32, 33] and publications that have been linked by UniProtKB or PubMed to the query sequence; a short summary assigns a confidence score to each of these metrics. While all analysis steps are run by default, it is possible to customize the pipeline to run only the processes required. A single plain text or FASTA protein sequence is required as the input to the tool and a species in which to identify possible orthologs in must be selected, making ORCAN very well suited to a complete analysis of a novel protein sequence, but less practical if the aim is to identify potential orthologs to multiple genes across a wide range of species.

OrthoList 2 is a resource for identifying orthologs between human and *C. elegans*. It integrates orthology calls from six different orthology prediction tools, five of which are also included in HCOP, with the resource absent from HCOP being OrthoInspector [34]. Like HCOP OrthoList 2 allows the use of a range of different gene identifiers, gene names or HGNC symbols as query terms; additionally, it can be searched with SMART IDs [35], InterPro domain IDs [36] or OMIM phenotypes [37]. Results are returned as a simple HTML table with links out to associated resources where appropriate. The number of orthology resources that contain the prediction is shown, but no ‘confidence score’ is calculated. Results can be exported as a CSV file. While OrthoList displays some additional content in terms of protein domain and phenotype information, the frequency at which OrthoList is updated, with 7 years between the releases of OrthoList 1 and Ortholist 2, means that the data it displays may quickly become out of date as the individual orthology, nomenclature, protein domain and human-disease phenotype resources release new data sets. Also of concern is the use of HGNC symbols without the accompanying HGNC IDs as one of the ways to identify the orthologous human genes. While the HGNC aims to minimize changes to gene symbols, their stability cannot be guaranteed and always recommends resources use symbols only alongside the stable HGNC ID.

To assess the coverage of the orthology predictions contained within HCOP, a comparison of the human–mouse HCOP ortholog pairs with those predicted by the Quest for Orthologs (QfO) 2018 set of benchmark data [38] was carried out. Despite significant differences in the source data between QfO and HCOP, approximately 50% of the predictions in HCOP were also made by one or more of the orthology resources in the QfO benchmark set. Of the 50% made only by HCOP, the majority of these predictions were supported by a single resource—most frequently OrthoMCL, which aims to identify ortholog groups rather than pairwise orthologs—and almost all of these predictions related to genes from complex gene families such as zinc finger proteins, olfactory receptors and keratin associated proteins. Some of the differences can also be explained by the QfO data being built using UniProt proteome data, meaning that the RNA predictions contained within HCOP were absent from the QfO predictions. Differences are also expected because the QfO data set was built in 2018 and a new mouse genome assembly (GRCm39) was released in 2020 and is being used by several of the resources contained within HCOP.

## Future plans

Currently, HCOP is human centric in nature, meaning that only orthology assertions between a human gene and its orthologous gene(s) in another species are displayed. In future, we would like to remove this limitation by importing orthology data from each of the orthology resources for assertions made between genes from all of the species covered. This would enable a user to search for HCOP results between cat and dog, for example. Moving to a less human centric approach would be beneficial for our efforts in naming genes across other vertebrate species, particularly for cases where gene families may have undergone significant expansion in certain taxa, for genes that have either been pseudogenized or deleted in human, or for genes that fall within an annotation gap in the human genome. For example, the *CUPIN1* genes named in horse (VGNC:84140), dog (VGNC:84141), cat (VGNC:84142) and cow (VGNC:84143) in VGNC are orthologs to the *CUPIN1P* pseudogene (HGNC:54490) in human, but as the automated gene naming system employed by VGNC relies on identifying one-to-one orthologs between protein coding genes in HCOP, the relationship between these genes was not automatically picked up and nomenclature was instead assigned manually by a VGNC curator. As the VGNC expands to cover new species, it may make more sense to use the taxonomically closest existing VGNC species as the starting point for automatically assigning nomenclature data, rather than basing everything on human data alone.

We will continue to periodically re-evaluate the data HCOP includes and will add any new species or orthology resources if appropriate. For orthology resources such as OrthoInspector where a software package to compute a chosen ortholog set is provided rather than precomputed data, we intend to assess if running and maintaining the software ourselves is feasible. We will investigate expanding the information displayed for an HCOP result, for example by displaying shared protein domain information between the ortholog pairs and incorporating functional annotation such as GO terms, as well as potentially adding links to selected publications for each gene. In the shorter term, a REST endpoint for HCOP data is currently in development as part of a larger restructuring of the REST services provided by genenames.org. This endpoint will enable a HCOP search to be run for a single gene at a time and will return results in JSON or XML format.

## Conclusions

HCOP is a useful tool that allows easy comparison of orthology data between human and other key species from a variety of sources. HCOP data are also used in multiple other popular resources such as Mouse Genome Informatics, the International Mouse Phenotyping Consortium [39], PomBase, The Rat Genome Database and the Chicken Gene Nomenclature Consortium. The recent reimplementation and modularization of the pipeline code have allowed for data expansion, both in terms of the species covered and orthology and nomenclature sources imported, while also enabling any changes made to the source data to be incorporated into the HCOP dataset quickly as part of a daily update process, meaning that HCOP orthology data should always be current. The modern web technologies utilized in the latest version of https://www.genenames.org allow the HCOP web interface to be highly responsive; when a query is submitted, results are returned and displayed using a RESTful process meaning that even complex queries that may return many orthology predictions will complete successfully. HCOP also provides a powerful way of computationally identifying consensus orthologs that can be assigned nomenclature in an automated manner. This approach could be expanded in future to infer gene function in newly annotated genomes.

**Key Points**

- HCOP aggregates, displays and simplifies searching of orthology assertions from a variety of resources.
- HCOP enables easy comparison of orthology data between human and other key species.
- Recent updates take advantage of modern web technologies and have allowed for the inclusion of data from additional species and orthology sources.

## Data Availability

All HCOP data are available from https://www.genenames.org. There are no restrictions for using the data.

## Notes

**Bethan Yates** is a scientific programmer in the HGNC. She is the maintainer of HCOP and the VGNC website and is the corresponding author for this paper.

**Kristian A. Gray** is a senior computer programmer within the HGNC project team. He designs, develops and maintains the HGNC website, tools and frameworks as a full stack developer.

**Tamsin E.M. Jones** has been a Gene Nomenclature Advisor at the HGNC since 2018. She works on harmonization of gene nomenclature across vertebrate species.

**Elspeth A. Bruford** is a Director of Research at the University of Cambridge. She is a PI and the group coordinator of the HGNC and has been working in the HGNC since 1998.
